# Optimization of Urea-EnFET Based on Ta_2_O_5_ Layer with Post Annealing

**DOI:** 10.3390/s110504562

**Published:** 2011-04-27

**Authors:** Cheng-En Lue, Ting-Chun Yu, Chia-Ming Yang, Dorota G. Pijanowska, Chao-Sung Lai

**Affiliations:** 1 Department of Electronic Engineering, Chang Gung University, 259 Wen-Hwa 1 st road, Kwei-Shan, Tao-Yuan, 333, Taiwan; E-Mail: yolue411@gmail.com (C.-E.L.); nan.fttt18@gmail.com (T.-C.Y.); 2 Device Section, Department of WAT and Devices, Inotera Memories Inc., 667 Fuhsing 3 rd Road, Hwa-Ya Technology Park, Kwei-Shan, Tao-Yuan, 333, Taiwan; E-Mail: charming2006@inotera.com; 3 Institute of Biocybernetics and Biomedical Engineering, Polish Academy of Sciences, Ul. Ks. Trojdena 4, 02-109 Warsaw, Poland; E-Mail: dpijanowska@ibib.waw.pl; 4 Biosensor Group, Biomedical Engineering Research Center, Chang Gung University, 259 Wen-Hwa 1 st road, Kwei-Shan, Tao-Yuan, 333, Taiwan

**Keywords:** urea, enzymatic field effect transistor (EnFET), ion sensitive field effect transistor (ISFET), tantalum pentoxide (Ta_2_O_5_), post N_2_ annealing

## Abstract

In this study, the urea-enzymatic field effect transistors (EnFETs) were investigated based on pH-ion sensitive field effect transistors (ISFETs) with tantalum pentoxide (Ta_2_O_5_) sensing membranes. In addition, a post N_2_ annealing was used to improve the sensing properties. At first, the pH sensitivity, hysteresis, drift, and light induced drift of the ISFETs were evaluated. After the covalent bonding process and urease immobilization, the urea sensitivity of the EnFETs were also investigated and compared with the conventional Si_3_N_4_ sensing layer. The ISFETs and EnFETs with annealed Ta_2_O_5_ sensing membranes showed the best responses, including the highest pH sensitivity (56.9 mV/pH, from pH 2 to pH 12) and also corresponded to the highest urea sensitivity (61 mV/pC_urea_, from 1 mM to 7.5 mM). Besides, the non-ideal factors of pH hysteresis, time drift, and light induced drift of the annealed samples were also lower than the controlled Ta_2_O_5_ and Si_3_N_4_ sensing membranes.

## 1. Introduction

Variation of human body fluid in even tiny concentrations can be critical for clinical diagnosis. To monitor the small changes in the early stage, an accurate and stable sensor is needed. Enzymatic field effect transistor (EnFETs) is one of the sensor platforms. The earliest report of EnFET was proposed by Janata and Moss in 1980 [1]. Subsequently, many biomarkers have been detected by EnFET, such as penicillin [1], urea [2], glucose [3], creatinine [4], *etc*. To fabricate the EnFET, specific enzyme is immobilized on the surface of the sensing membrane of an ion sensitive field effect transistor (ISFET). Therefore, the sensing properties are not only dependent on the enzymatic layer but also dominated by the sensing membrane of ISFETs. For EnFET applications, Si_3_N_4_ layers deposited by low pressure chemical vapor deposition (LPCVD) are the most common sensing membranes of ISFETs because of the high sensitivity and good stability.

Until now, plenty of materials have been applied for the sensing membranes of pH ISFETs, including: SiO_2_, Si_3_N_4_ [5,6], Ta_2_O_5_ [7–9], Al_2_O_3_ [10], TiO_2_ [11,12], HfO_2_ [13,14], SnO_2_ [15], *etc*. In these materials, Ta_2_O_5_ layer has the largest surface buffer capacity (*β*), which gives the sensor better immunity to interference ions; wide applicable pH range, and high chemical resistance for the cleaning-in-place (CIP) test [14,16,17]. Moreover, the Ta_2_O_5_-ISFETs were also used as the platform of EnFETs [18,19]. However, the serious effect of light induced drift was observed on the ISFETs with Ta_2_O_5_ layers [20,21]. This drawback limited the applicable range of Ta_2_O_5_ sensing membrane, and many research groups are using different fabrication process and post annealing treatment to solve this problem.

In this paper, urea concentration was monitored by a pH ISFET based EnFET. The urea hydrolysis reaction by urease is as follows:

(1) $$\text{CO}{\left({\text{NH}}_{2}\right)}_{2}+3{\text{H}}_{2}\text{O}\overset{\text{urease}}{\to }{\text{CO}}_{2}+2{\text{NH}}_{4}^{+}+2{\text{OH}}^{-}$$

The sensing membrane of the pH ISFET was the Ta_2_O_5_ layer deposited by reactive radio frequency (rf) sputter system. In order to improve the sensing properties, the Ta_2_O_5_ layer was treated by post N_2_ annealing at 900 °C for 30 min. In addition, the sensing properties were also compared with the urea-EnFET with conventional Si_3_N_4_ sensing membrane.

## 2. Experimental

### 2.1. Chemicals

The pH sensing properties were measured to evaluate the pH-ISFETs. The phosphate buffer solutions were prepared with 5 mM NaH_2_PO_4_, 0.1 M NaCl, and deionized (DI) water as the background solution. The initial pH value was 5.7. Then, the pH value of the buffer solutions were adjusted by adding 0.1 M NaOH and 0.1 M HCl solutions with autoburettes (Mettler-Toledo) and monitored by a combined pH glass electrode.

For the urea detection, all solutions were purchased from Sigma, including urease (E.C. 3.5.1.5, type IX, at activity 20 kU), glutaraldehyde (GA), 3-aminopropyl-triethoxysilane (APTS), and urea (U5378, 98+%). Urea and urease solutions were diluted with 5 mM phosphate buffer, which has been adjusted to pH 6 as background because of the high sensitivity for urea detection [2].

### 2.2. Device Fabrication

**Figure 1.**
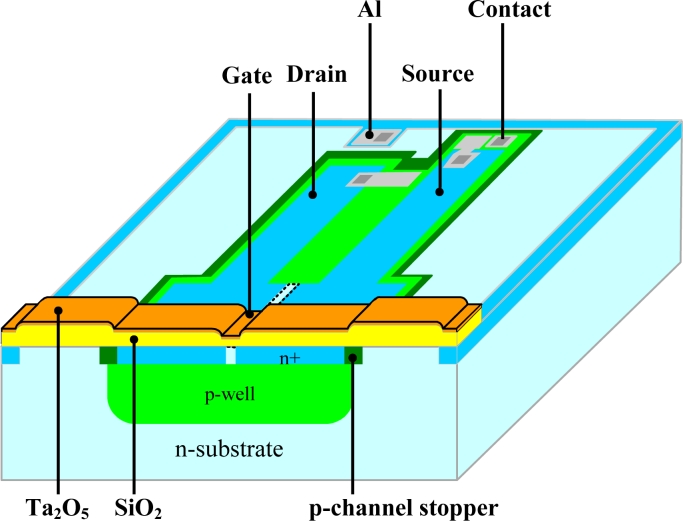
The structure and cross section of Ta_2_O_5_-ISFET.

The ISFETs were fabricated by Institute of Electron Technology (ITE, the polish abbreviation), Poland. The structure and cross section are shown in Figure 1. The ISFETs are designed with *p*-well arrangement on 3 inch (100) *n*-type wafers. The ratio of channel width and length was W/L = 600/16 (in μm). The 50 nm-thick buffering oxide layers between silicon and sensing membrane were deposited by dry oxidation. The sensing membranes, Ta_2_O_5_ layers, were deposited by rf sputtering in Chang-Gung University (CGU), Taiwan. For the rf sputtering of Ta_2_O_5_ layers, a pure Ta target was used. The rf power was fixed at 150 W, and the process pressure was 10 mTorr. The Ar/O_2_ gas mixture ratio was controlled by mass flow controllers (MFC) as 22.5/2.5 in sccm. To improve the sensing membrane, a post deposition annealing (PDA) in N_2_ ambient at 900 °C for 30 min was used. For the control group, the ISFETs with Si_3_N_4_ sensing membrane were prepared. The Si_3_N_4_ layers were deposited by LPCVD using the standard process parameters of ITE [2,22,23].

Finally, all ISFETs were assembled on printed circuit boards (PCB) with a silver paste (TED PELLA, Inc.) and then encapsulated with epoxy resin type adhesive JU-100 (KOKI Company Ltd.) with open windows of 3 × 3 mm^2^.

### 2.3. Enzyme Immobilization

**Figure 2.**
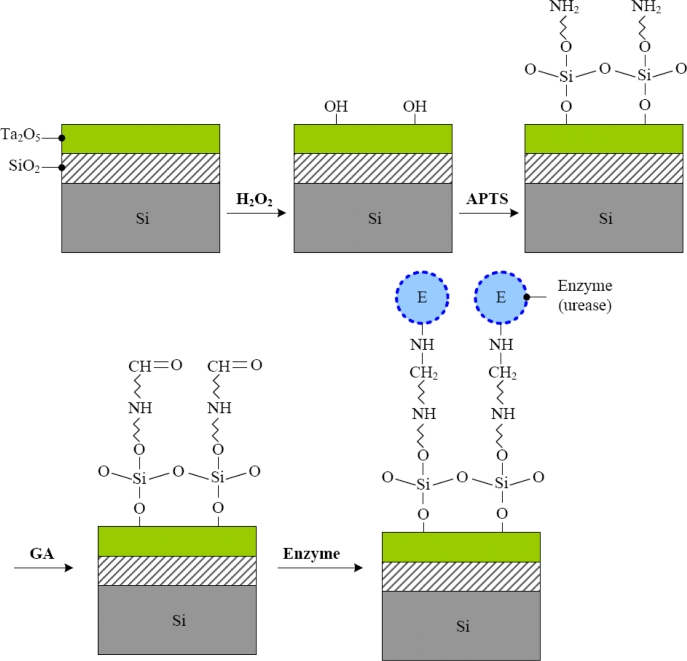
Schematic of covalent bonding process flow of urea-EnFET based on Ta_2_O_5_ sensing membrane.

The covalent bonding steps are shown in Figure 2. At first, to generate hydroxyl bond, the encapsulated Ta_2_O_5_-ISFETs were immersed into 10% hydrogen peroxide (H_2_O_2_) solution for 24 hours. Afterwards, the samples were put into 40 °C, 9% APTS solution for 4 hours for silylation. Then, the samples were immersed into 10% of glutaraldehyde for 1 hour, which is a dual function group to crosslink amine bond on urease and APTS. Finally, the urease powder was mixed with the phosphate buffer as the concentration of 1.5 μg/mL, and then 20 μL-urease was dripped on the open window of ISFET before storing the sample at 4 °C (fridge) overnight. After rinsing non-immobilized enzyme by phosphate buffer, the EnFETs were ready for measurement.

### 2.4. Measurement Setup

To evaluate the performance of ISFETs and EnFETs, the drain-to-source current *vs.* gate-to-source voltage (I_DS_–V_GS_) curves were measured by HP 4156, firstly. Afterwards, a constant drain-source voltage and constant drain-source current (CVCC) circuit was used in measurements with I_DS_ = 100 μA and V_DS_ = +0.5 V. To evaluate the pH sensing properties, the commercial pH buffer solutions from pH 2 to pH 12 were used. For the urea sensitivity, different urea concentrations from 1 mM to 10 mM were prepared by diluting 5 mM phosphate buffer at pH 6. All of the measurements were done at 25 °C.

In addition, for all the measurement, a commercial Ag/AgCl electrode was used to provide the sensing system a constant and stable reference potential.

## 3. Results and Discussion

**Figure 3.**
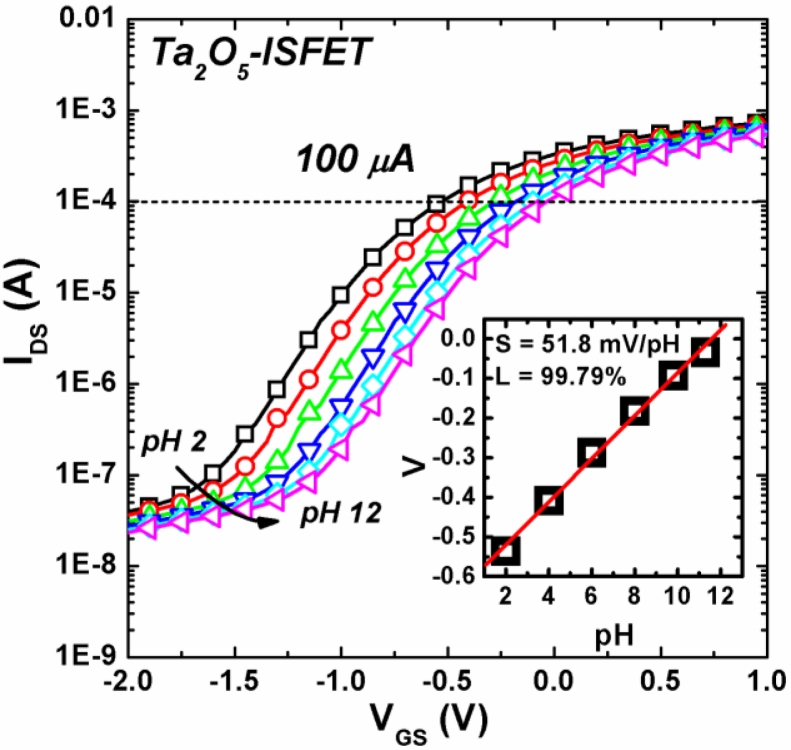
I_DS_–V_GS_ curves of Ta_2_O_5_-ISFET measured in the buffer solutions with different pH value (from pH 2 to pH 12).

The I_DS_–V_GS_ curves of Ta_2_O_5_-ISFET measured in the buffer solutions from pH 2 to pH 12 are shown in Figure 3. The curves shifted to the right-side when the ISFETs were measured in buffer solutions of high pH values. The output voltage of different pH buffer solution was calculated from the corresponding point at I_DS_ = 100 μA. The output voltage and calibration curve are shown in the insert of Figure 3. For this Ta_2_O_5_-ISFET without post treatment, the pH sensitivity was 51.8 mV/pH with a high linearity (99.79%) of the calibration curve.

Afterwards, the sensing properties including sensitivity, hysteresis, and drift were measured by the CVCC circuit. The results of Ta_2_O_5_-ISFETs without and with post annealing are listed in Table 1. Besides, the results of standard Si_3_N_4_-ISFET are also included. In this table, both Ta_2_O_5_-ISFETs without and with post annealing show higher sensitivity than Si_3_N_4_-ISFET. Especially for the ISFETs with annealed Ta_2_O_5_ sensing membrane, a near Nernstian response of 56.9 mV/pH was performed. In addition, better reliability was observed from the low hysteresis (with the measurement loop of pH 6-2-6-12-6, and 6-12-6-2-6) and drift (measured in pH 4 for 12 hours). The improved sensing properties of ISFETs with Ta_2_O_5_ layer could be caused by the formation of polycrystalline, the increase of surface area, and the increasing of oxygen density [13,24,25].

**Figure 4.**
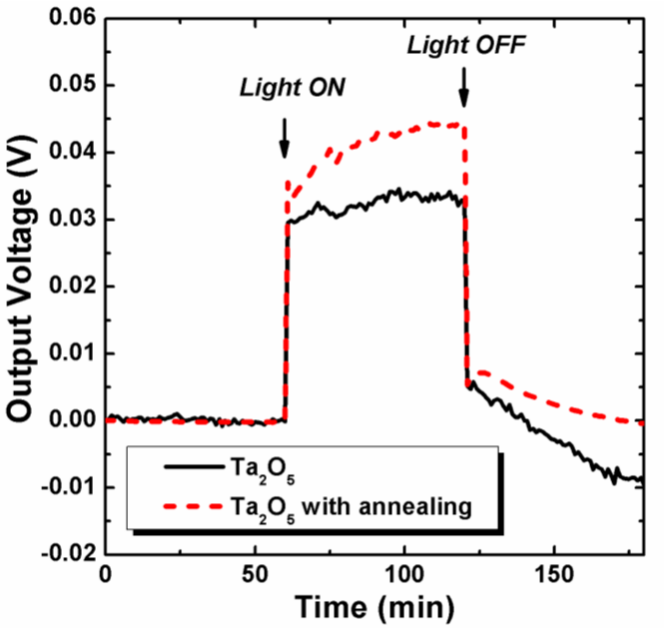
Light induced drift of the Ta_2_O_5_ ISFETs without and with post annealing.

In addition, the light induced drift of the Ta_2_O_5_ ISFETs without and with post annealing was measured with a light source of optical microscope. The light intensity was 4,500 lux measured by a light meter. The ISFETs were stored in a beaker covered with aluminum foil for the uniform light illumination for all ISFETs. The V_GS_ of ISFETs were firstly measured under dark condition for 1 hour, then with the light turned on for 1 hour, and then back under the dark condition for 1 hour. The results are shown in Figure 4. During the illumination, both curves shifted to the higher potential, similar to drift phenomena, which could be resulted by the light generated electrons and holes in the dielectric layer and silicon substrate [9]. The curves of Ta_2_O_5_-ISFETs with post annealing showed better stability. Besides, after illuminating and back to the dark condition, the output voltage returned to the initial output voltage.

**Figure 5.**
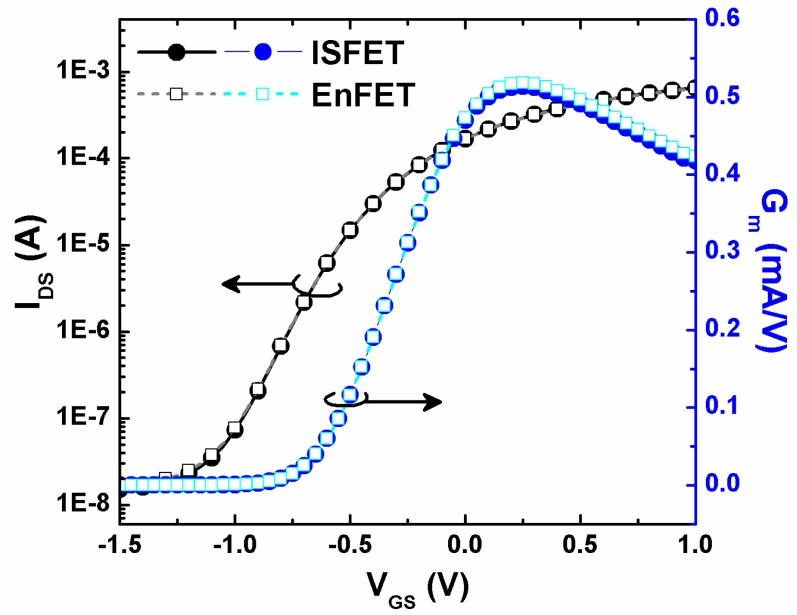
I_DS_–V_GS_ curves and transconductance of ISFET and EnFET.

After the evaluation of the pH sensing properties of the test ISFETs, other ISFETs were processed for the covalent bonding and urease immobilization. For monitoring the urea concentration by EnFET, at first, the I_DS_–V_GS_ curves and transconductance (G_m_) of Ta_2_O_5_-ISFET and EnFET were measured by HP 4156, as shown in Figure 5. The similar response of ISFET and EnFET means the surface capacitance had almost no change before and after the chemical reaction of covalent bonding and urease immobilization. The good electrical compatibility between ISFET and EnFET are very suitable for the further application of enzymatic EnFET/ISFET (REFET) pair [26].

**Figure 6.**
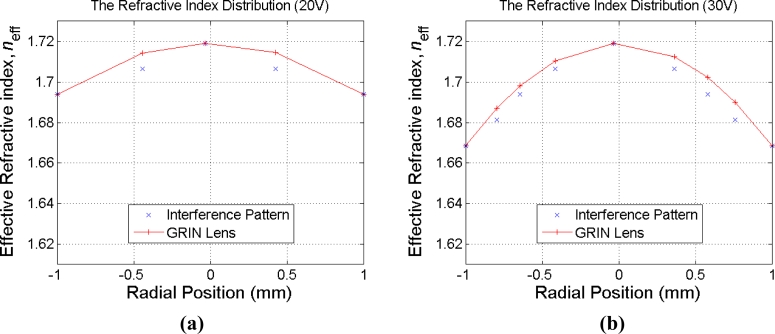
Time response to output voltage measured in 0–30 mM urea.

The time response to output voltage measured in 0–30 mM urea are shown in Figure 6. In this figure, the output voltages were stable in about 2 min. In addition, the suitable operation region of urea concentration could also be observed in this figure. The response could be divided to three regions: (I) Low response (lower than 1 mM), which could be due to the limitation of the lowest urea concentration, (II) High response (between 1 mM to 15 mM), which showed the higher voltage shift and could be the suitable region for urea detection, (III) Saturation region (higher than 15 mM), where very low voltage shift was observed even though the urea concentration increased.

**Figure 7.**
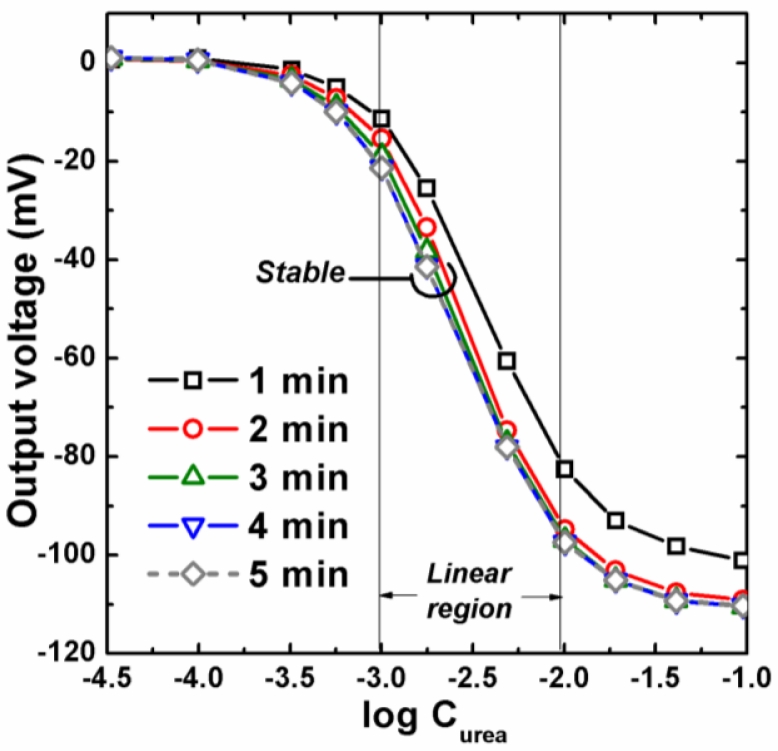
Calibration curves of output voltage with 1–5 min sampling time.

Based on Figure 6, the calibration curves of output voltage with different sampling time were also replotted and shown in Figure 7. In this figure, the calibration curves sampled from different time were checked again, which showed only slight shift between 2 and 5 min. Concerning time and accurate response, 5 min sampling time was chosen for further evaluation. In addition, the suitable operating region for urea detection by EnFET was redefined as logC_urea_ from −3.00 (1 mM) to −2.12 (7.5 mM), which was caused by the change of the unit of urea concentration from linear scale to log scale.

**Figure 8.**
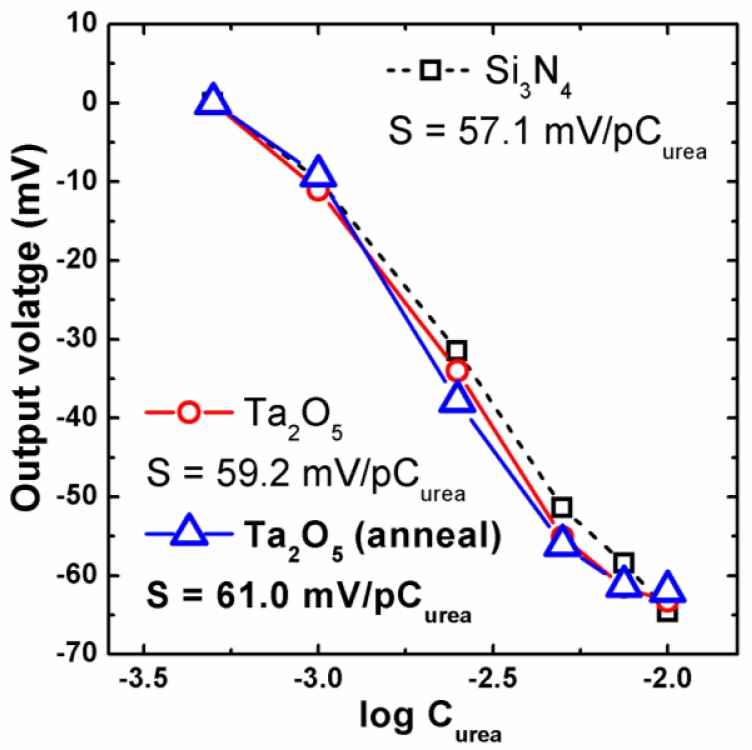
Calibration curves of the urea-EnFETs with Si_3_N_4_, Ta_2_O_5_, and annealed Ta_2_O_5_ sensing membranes.

Finally, the calibration curves of the urea-EnFETs with Si_3_N_4_, Ta_2_O_5_, and annealed Ta_2_O_5_ sensing membranes were compared in Figure 8. For all samples, the linearity is higher than 99% between 1 mM to 7.5 mM of urea. The sensitivity of EnFET with Si_3_N_4_ sensing membrane is 57.1 mV/pC_urea_, and the sensitivity for the EnFETs with Ta_2_O_5_ and annealed Ta_2_O_5_ sensing membranes are 59.2 mV/pC_urea_ and 61.0 mV/pC_urea_, respectively. Since the urea detection is based on the increase of the byproduct of hydrogen ions, the sensitivity of EnFETs for urea concentration is also dependent on the pH sensitivity of ISFETs. In this case, the ratio of pH sensitivity and urea sensitivity is about 1.1 ± 0.43.

## 4. Conclusions

The urea-EnFET with covalent bonding process was investigated based on the pH-ISFETs with Ta_2_O_5_ sensing membranes. In addition, the sensing properties of Ta_2_O_5_-ISFETs were also improved from the control samples by the post N_2_ annealing, including higher pH sensitivity, lower hysteresis, lower drift coefficient, and lower light induced drift. The improved sensing properties were also corresponding to the EnFETs. In this research, the output responses of EnFETs were stabilized in 2 min, and the applicable and linear measurement range for urea concentration was from 1 mM to 7.5 mM. For the EnFET with annealed Ta_2_O_5_ sensing membrane, the sensitivity was 61 mV/pC_urea_. Based on the high sensitivity and minimized non-ideal factors, the Ta_2_O_5_ layer with post annealing could be used to replace the conventional Si_3_N_4_ layer as the basic substrate platform for biomedical applications, such as chemically modified field effect transistors (ChemFETs), EnFETs with other enzyme, and DNA-FETs.

## Figures and Tables

**Table 1.** The sensing properties of ISFETs with Si_3_N_4_, Ta_2_O_5_, and annealed Ta_2_O_5_ sensing membranes.

	pH sensitivity (mV/pH)	Hysteresis (6-2-6-12-6) (mV)	Hysteresis (6-12-6-2-6) (mV)	Drift coefficient (mV/h)
				
Si_3_N_4_	51.1	—	5.1	<1 mV/h
Ta_2_O_5_	51.8	15.2	6.3	<1 mV/h
Ta_2_O_5_ (anneal)	56.9	0.3	0.7	<1 mV/h
